# Increased mRNA expression for serotonin receptor 1B (*HTR1B*) is associated with thrombosis in *BCR::ABL1*‐negative myeloproliferative neoplasms

**DOI:** 10.1111/jcmm.70024

**Published:** 2024-08-25

**Authors:** Petruta Gurban, Cristina Mambet, Anca Botezatu, Laura G. Necula, Lilia Matei, Ana Iulia Neagu, Ioana Madalina Pitica, Laura Denisa Dragu, Alina Nastasie Schulman, Marius Ataman, Saviana Nedeianu, Mihaela Chivu‐Economescu, Coralia Bleotu, Gabriela Anton, Carmen Cristina Diaconu

**Affiliations:** ^1^ Cellular and Molecular Pathology Department, Stefan S. Nicolau Institute of Virology Romanian Academy Bucharest Romania; ^2^ Cytogenomic Medical Laboratory Bucharest Romania; ^3^ Department of Radiology, Oncology, and Hematology, Faculty of Medicine Carol Davila University of Medicine and Pharmacy Bucharest Romania; ^4^ Hematology Department Emergency University Clinical Hospital Bucharest Romania; ^5^ Molecular Virology Department Stefan S. Nicolau Institute of Virology, Romanian Academy Bucharest Romania; ^6^ Department of Infectious Diseases, Epidemiology, Microbiology, Parasitology, Virology, Diabetes, Endocrinology, Faculty of Medicine Carol Davila University of Medicine and Pharmacy Bucharest Romania

**Keywords:** *BCR::ABL1*‐negative myeloproliferative neoplasms, *JAK2* V617F mutation, major thrombosis, microvascular thrombosis, serotonin receptor 1B (*HTR1B*) gene

## Abstract

*BCR::ABL1*‐negative myeloproliferative neoplasms (MPNs) are clonal haematopoietic stem cell disorders characterized by specific driver mutations and an increased risk of both macrothrombosis and microthrombosis. Serotonin receptor type 1B (HTR1B) was found to be expressed by various solid tumours, and also primary bone marrow mononuclear cells from myelodysplastic neoplasm and acute myeloid leukaemia patients, representing a potential therapeutic target. In this study we assessed for the first time the expression levels of *HTR1B* mRNA in the peripheral blood mononuclear cells (PBMC) of 85 newly diagnosed MPN patients, consisting of 28 polycythemia vera, 25 essential thrombocythemia and 32 primary myelofibrosis cases. Levels of *HTR1B* expression between MPN subtypes and control group were not significantly different. However, at clinical data examination, it was observed that MPN patients with a recent history of major thrombosis and/or signs of impaired microcirculation exhibited significantly higher *HTR1B* expression levels compared to non‐thrombotic MPNs and control group. Moreover, thrombotic MPN patients had significantly higher *HTR1B* expression than patients with recent thrombosis and absence of MPN diagnostic criteria. These findings suggest that increased levels of *HTR1B* expression in PBMC might be associated with thrombosis in MPN patients, but larger studies are needed for confirmation, including testing of the receptor protein expression level.

## 1 INTRODUCTION

5‐Hydroxytryptamine (5‐HT), or serotonin, acts both as a neurotransmitter in the central nervous system and as a peripheral regulatory hormone, being involved in a wide spectrum of physiological functions.^1^ The enterochromaffin cells of the gut synthesize most of the peripheral 5‐HT and released it into the bloodstream^2^—where it is captured by platelets (PLTs) through plasma membrane serotonin transporter.^3^ PLTs store 5‐HT in the dense granules, being secreted upon their activation.^4^ The biological effects of 5‐HT are mediated by seven families of receptors (5‐HTR1‐7), most of them being G‐protein coupled receptors that are selectively expressed by various cells.^5^ The HTR1 family is further divided in five receptor subtypes (HTR1A, HTR1B, HTR1D, HTR1E and HTR1F) that connect with intracellular Gαi/Gαo proteins, suppressing adenylyl cyclase activity.^6^

As shown by different preclinical studies, serotonin displays carcinogenic properties, and together with 5‐HT receptors was involved in tumour growth, angiogenesis, invasion and metastasis,^7^ affecting cancer cells, as well as immune and stromal cells from the tumour microenvironment.^8^ Moreover, some tumour tissues express various subtypes of 5‐HTR, through which serotonin exerts a mitogenic effect, inhibiting malignant cell apoptosis due to the activation of MAPK and PI3K/Akt signalling pathways.^8^ Particularly, serotonin receptor type 1B (HTR1B) is expressed by cell lines and/or primary cells from prostate, breast, colorectal, hepatocellular, pancreatic, ovarian, lung and urinary bladder cancer tissues.^8^

Concerning haematological malignancies, it was previously shown that *HTR1A* and *HTR1B* were overexpressed at RNA level in bone marrow mononuclear cells obtained from patients diagnosed with acute myeloid leukaemia (AML), compared to mature cells and the most primitive haematopoietic cell fraction isolated from healthy donors.^9^ Inhibition of HTR1 signalling induced both terminal differentiation and apoptosis, the effect being more pronounced in leukaemia stem cells (LSCs) versus more mature AML blasts, while a minimal effect was observed on healthy blood cells.^9^ These findings indicated the importance of HTR1 receptors in leukemogenesis making them a potential therapeutic target in AML, especially for eradicating LSCs responsible for disease recurrence.^9^ HTR1B had been proposed as a therapeutic target in myelodysplastic syndrome (MDS), as well as in chronic myelomonocytic leukaemia (CMML). HTR1A/B exhibited an increased expression in primary bone marrow or mononuclear cells from MDS and CMML patients, compared to samples from the control group, based on surface phenotype cytometric analysis.^10^

As opposed to AML, *BCR::ABL1*‐negative myeloproliferative neoplasms (MPNs) are clonal disorders of haematopoietic stem cells (HSCs) with a chronic disease course, that comprise three classical subtypes: polycythemia vera (PV), essential thrombocythemia (ET), and primary myelofibrosis (PMF).^11^ From the point of view of molecular pathogenesis, MPNs are characterized by specific somatic driver mutations in one of three genes, Janus Kinase 2 (*JAK2*), Calreticulin (*CALR*), *thrombopoietin receptor* (*MPL*), that induce a constitutive Janus kinase–signal transducer and activator of transcription (JAK/STAT) signalling.^12^

Given the previous reports of overexpression of HTR1B in AML and MDS, and that MPNs share certain epigenetic mutations with these entities, we assessed the expression levels of *HTR1B* mRNA in patients with MPNs compared to healthy subjects. We aimed to observe differences in expression according to MPN subtype. We did not find significant differences between MPN and control samples with respect to *HTR1B* expression, however, we found an increased expression in mononuclear cells of the thrombotic‐MPN patients compared to patients with non‐MPN thrombosis and that may have impact in prediction of MPNs thrombotic complications.

## 2 MATERIALS AND METHODS

### 2.1 Patients and samples

The current study was performed retrospectively on samples stored in the biobank of Stefan S Nicolau Institute of Virology, Romania. The samples were previously obtained from newly diagnosed MPN patients, according to 2016 World Health Organization criteria,^13^ that were referred to the institute for molecular tests. Cases were selected from different Haematology Units based on MPN phenotype and availability of blood cell fractions in the biobank. A complete medical record was provided for all cases. Additionally, healthy volunteers without anomalies at automated complete blood count (CBC) were included as controls for the comparative analysis. In a subsequent step, samples from non‐MPN patients that presented recent history of major thrombosis were also analysed. The ethical approval was obtained from the local ethics committee (No. 136/06.02.2017 rev. no. 131/18.01.2019) and the research was conducted in conformity with the Declaration of Helsinki. A written informed consent was obtained from each subject at collection of blood samples.

Peripheral mononuclear cells (PBMC) were separated by density gradient centrifugation using Ficoll‐Paque medium, (GE Healthcare Life Sciences, USA), and granulocytes were isolated after PBMC removal and red blood cell lysis of the remained pellet in hypotonic solution.

DNA was extracted from the cell fractions with PureLink™ Genomic DNA Mini Kit (ThermoFisher Scientific, USA). Total RNA was extracted from PBMC using PureLink™ RNA Mini Kit (ThermoFisher Scientific, USA), following the manufacturer's instructions. High‐Capacity cDNA Reverse Transcription Kit (ThermoFisher Scientific, USA) was employed for the reverse transcription reaction.

### 2.2 MPN‐driver mutations genotyping

MPN driver mutations were genotyped in peripheral blood granulocytes using previously described methods: allele‐specific PCR for *JAK2* V617F mutation,^14^ bidirectional Sanger sequencing for *CALR* exon 9 mutations,^15^ and Real‐Time PCR with Ipsogen MPL W515L/K MutaScreen Kit (Qiagen, USA) for *MPL* W515L/K mutations.

### 2.3 *JAK2* V617F mutational load quantification

*JAK2* V617F mutational load was measured in granulocyte DNA samples using Ipsogen JAK2 MutaQuant Kit (Qiagen) on Applied Biosystems™ 7500 Real‐Time PCR System (ThermoFisher Scientific, USA). The copy number of each *JAK2* allele (wild‐type and mutant) was calculated from the standard curves with SDS software version 1.4.1 (Thermo Fisher Scientific, USA). *JAK2* V617F mutational load was expressed as the percentage of *JAK2* mutant allele copy number of the total number of *JAK2* copies.

### 2.4 Real‐time quantitative PCR (qPCR) for *HTR1B* gene expression

*HTR1B* gene expression analysis was performed on Applied Biosystems™ 7500 Real‐Time PCR System (ThermoFisher Scientific, USA). An amount of 50 ng of cDNA per sample was introduced into the PCR reaction together with TaqMan™ Fast Universal PCR Master Mix (2X), no AmpErase™ UNG (ThermoFisher Scientific, USA), and the mixture of specific primers and HTR1B‐FAM‐labelled probe (Hs_00265286, ThermoFisher Scientific, USA). *GAPDH* was used as an internal control to normalize mRNA levels. Expression of the reference gene in cDNA samples was determined with Applied Biosystems™ Human GAPD (GAPDH) Endogenous Control (VIC™/TAMRA Probe, Primer Limited), containing the mixture of specific primers and VIC‐TAMRA‐labelled oligonucleotide probe.

For qPCR data normalization the double delta Ct (∆∆Ct) method was used. The housekeeping gene GAPDH was considered as reference. To obtain the fold change in *HTRB1* gene expression levels relative to control 2^−∆∆Ct^ was calculated. Results were displayed as log_10_ fold change.

### 2.5 Statistical analysis

Statistical analysis was performed using version 8.0.1 of GraphPad Prism software (GraphPad, San Diego, USA). The non‐parametric Kruskal–Wallis test followed by post‐hoc Dunn's multiple comparisons test were used to assess differences in continuous variables among more than two groups of subjects, while the non‐parametric Mann–Whitney test was employed for evaluating the differences between two groups. In case of qualitative variables Chi‐square test or Fisher's exact test were used for the comparative analysis. *p* < 0.05 were considered statistically significant.

## 3 RESULTS

### 3.1 Clinical characteristics, haematological parameters and driver mutation status across MPN subtypes

A total number of 85 newly diagnosed MPN patients was included in the study, divided into 28 PV, 25 ET and 32 PMF cases, according to the disease subtype (Table 1). No significant differences in demographic data and white blood cell (WBC) count were observed between MPN subtypes. As expected, PV patients exhibited significantly higher haemoglobin (Hb) values, and ET patients a higher PLT count. The frequency of MPN driver mutations was not significantly different among the three groups of patients, except for *CALR* mutations that displayed a higher frequency in the PMF group. Two MPN patients that did not harbour mutations in any of the three genes were classified as ‘triple negative’ (Table 1).

**TABLE 1:** Demographic data, haematological parameters and profile of driver mutations of MPN patients stratified by disease subtype.

Variables	All patients (*n* = 85)	PV (*n* = 28)	ET (*n* = 25)	PMF (*n* = 32)	*p*‐value
Age, median (range), years	57 (23–80)	49.5 (23–78)	59 (24–80)	58 (26–77)	0.3549
Gender (male), *n* (%)	42 (49.4)	16 (57.1)	12 (48.0)	14 (43.7)	0.8325
WBC, median (range), × 10^9^/L	9.6 (5.2–38.1)	9.6 (5.7–23.6)	8.6 (6.3–13.85)	10.2 (5.2–38.1)	0.5202
Hb, median (range), g/dL	14.2 (8.4–23.3)	16.8 (14.1–23.3)	14.5 (12.2–16.2)	12.3 (7.4–16.1)	**<0.0001**
PLT, median (range), × 10^9^/L	638 (34–1800)	594 (308–1800)	831 (443–1568)	572.5 (34–1800)	**0.0031**
MPN driver mutation status					
*JAK2* V617F, *n* (%)	69 (81.2)	28 (100)	20 (80.0)	21 (65.6)	0.5518
*CALR* exon 9, *n* (%)	11 (12.9)	0 (0)	2 (8.0)	9 (28.1)	**0.0142**
*MPL* exon 10, *n* (%)	3 (3.5)	0 (0)	2 (8.0)	1 (3.1)	0.3145
Triple‐negative	2 (2.3)	0 (0)	1 (4.0)	1 (3.1)	0.6021

Abbreviations: ET, essential thrombocythemia; Hb, haemoglobin; MPN, myeloproliferative neoplasm; PLT, platelets; PMF, primary myelofibrosis; PV, polycythemia vera; WBC, white blood cells.

Bold values denote statistical significance at the (*p* < 0.05) level.

### 3.2 *HTRB1* gene expression levels in different MPN subtypes and control group

For the comparative analysis of *HTR1B* gene expression levels a number of six healthy volunteers without any anomaly of automated CBC results were included in the study.

**FIGURE 1:**
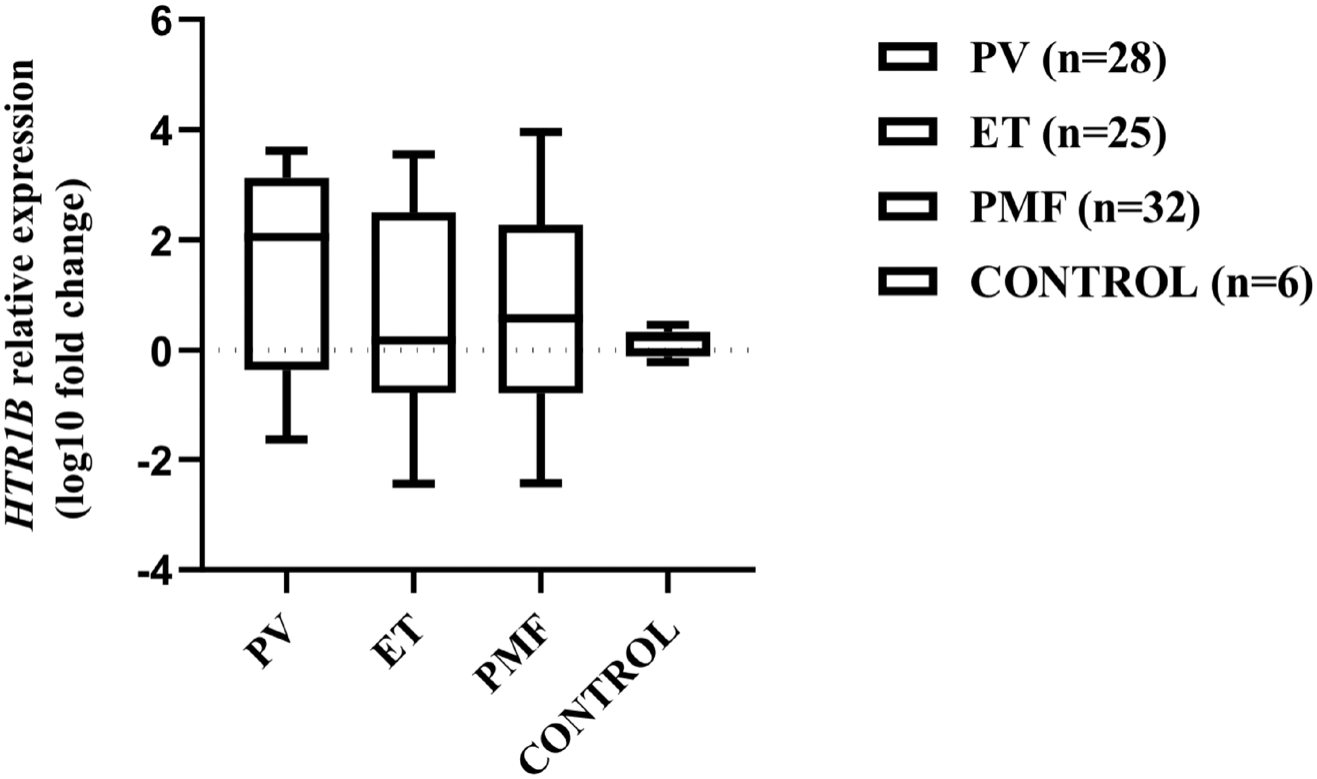
*HTR1B* mRNA relative expression levels in patients with different MPN subtypes and control group. Each box plot indicates the median, interquartile range, minimum and maximum log_10_ fold change values.

When comparing the levels of *HTR1B* expression between MPN subtypes and the control group, no statistically significant difference was detected (*p* = 0.3089, Kruskal–Wallis test) (Figure 1).

### 3.3 *HTRB1* gene expression analysis in relation to thrombotic events

**FIGURE 2:**
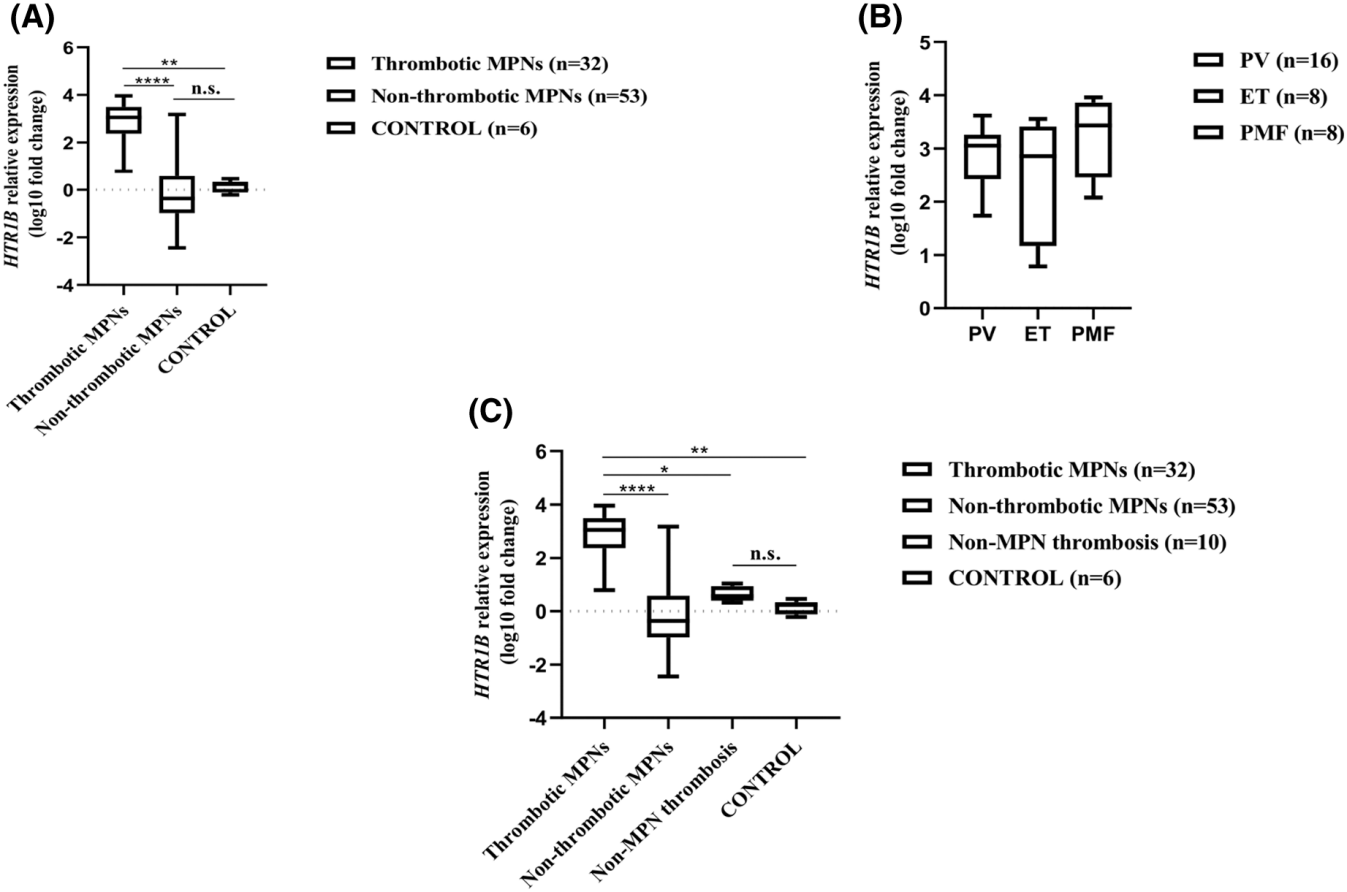
*HTR1B* mRNA relative expression levels in relation to thrombotic events. (A) *HTR1B* mRNA relative expression levels in thrombotic MPNs, non‐thrombotic MPNs and control group. (B) *HTR1B* mRNA relative expression levels within the MPN thrombotic group stratified by MPN subtype. (C) *HTR1B* mRNA relative expression levels in thrombotic MPNs, non‐thrombotic MPNs, non‐MPN thrombotic group and control group. Each box plot indicates the median, interquartile range, minimum and maximum log_10_ fold change values; *p < 0.05, **p < 0.01, ****p < 0.0001, n.s. not significant.

Due to the large variation in fold change values across MPN subtypes, we analysed the clinical data of MPN patients and observed highly significant increased levels of *HTR1B* expression in patients who either had at least one episode of major thrombosis (ischemic stroke, deep vein thrombosis with or without pulmonary embolism, splanchnic vein thrombosis) at the time of diagnosis or on the recent history, or showed signs of impaired microcirculation (headache, paresthesia, erythromelalgia) possibly due to microvascular thrombosis, as considered previously.^16^ Further on, MPN patients were divided into thrombotic and non‐thrombotic groups, and their clinical characteristics, CBC results, driver mutation status and *JAK2* V617F mutational load are displayed in Table 2, without significant differences between the two MPN groups. However, there was a statistically significant difference in the fold change of *HTR1B* expression between thrombotic MPNs, non‐thrombotic MPNs and control group (*p* < 0.0001, Kruskal–Wallis test) (Figure 2A). At post‐hoc Dunn's multiple comparisons test thrombotic MPN patients exhibited significantly higher *HTR1B* relative expression levels compared to non‐thrombotic MPNs (median log_10_ fold change 3.055 vs. −0.3649, *p* < 0.0001), as well as to control group (median log_10_ fold change 3.055 vs. 0.08451, *p* = 0.0041) (Figure 2A). Within the thrombotic MPN group there was no statistically significant difference between the three MPN subtypes in respect to the fold change of *HTR1B* expression.

**TABLE 2:** Clinical characteristics, haematological parameters, driver mutation status, and *JAK2* V617F mutational load of thrombotic and non‐thrombotic MPN patients.

Variables	Thrombotic MPNs (*n* = 32)	Non‐thrombotic MPNs (*n* = 53)	*p‐*value
Age, median (range), years	57 (23–78)	57 (23–80)	0.4855
Gender (male), n (%)	17 (53.1)	25 (47.2)	0.7580
WBC, median (range), × 10^9^/L	9.1 (5.7–33.5)	9.7 (5.2–38.1)	0.6500
Hb, median (range), g/dL	14.7 (7.4–23.3)	14 (8.4–20.9)	0.2388
PLT, median (range), × 10^9^/L	720.5 (189–1800)	597.5 (34–1395)	0.1585
MPN subtype			
PV	16 (50.0)	12 (22.6)	0.0723
ET	8 (25.0)	17 (32.1)	0.6559
PMF	8 (25.0)	24 (45.3)	0.1986
Major thrombosis, *n* (%)	24 (75.0)	–	
Arterial, *n* (%)	16 (50.0)	–	
Venous, *n* (%)	8 (25.0)	–	
Vasomotor (microvascular) disturbances, *n* (%)	8 (25.0)	–	
MPN driver mutation status			
*JAK2* V617F, *n* (%)	29 (90.6)	40 (75.5)	0.5803
*JAK2* V617F%, median (range)	30.3 (12.7–100)	40.6 (11.6–92.8)	0.1607
*CALR* exon 9, *n* (%)	2 (6.2)	9 (17)	0.2040
*MPL* exon 10, *n* (%)	1 (3.2)	2 (3.8)	0.8794
Triple‐negative	0 (0)	2 (3.8)	0.2751

Abbreviations: ET, essential thrombocythemia; Hb, haemoglobin; MPN, myeloproliferative neoplasm; PLT, platelets; PMF, primary myelofibrosis; PV, polycythemia vera; WBC, white blood cells.

(*p* = 0.2448, Kruskal–Wallis test) (Figure 2B).

To investigate whether the increased *HTR1B* expression was a feature of thrombotic MPN patients or was due to the presence of the thrombotic process itself, unrelated to MPN, we assessed *HTR1B* expression in PBMCs obtained from 10 patients with recent major thrombotic episodes (four cases of portal vein thrombosis, two cases of stroke in patients under 55 years, one case of stroke in a diabetic and hypertensive patient, one case of cerebral vein thrombosis, one case of posttraumatic subclavian vein thrombosis, one case of venous thromboembolism in association with colon adenocarcinoma), exhibiting normal CBC parameters and negative results at MPN driver mutations tests. When comparing the level of *HTR1B* expression between MPN patients with/without thrombosis, non‐MPN thrombotic patients and control group a statistically significant difference was noted (*p* < 0.0001, Kruskal–Wallis test) (Figure 2C). At post‐hoc Dunn's multiple comparisons test thrombotic MPN patients exhibited significantly higher *HTR1B* relative expression levels compared to non‐MPN thrombotic patients (median log_10_ fold change 3.055 vs. 0.5608, *p* = 0.0253), while the transcript expression levels were not significantly different between non‐MPN thrombotic patients and control group (median log_10_ fold change 0.5608 vs. 0.08451, *p* = 0.9999) (Figure 2C).

## 4 DISCUSSION

The results obtained indicated that MPN patients who had at least one major thrombotic episode and/or signs of impaired microcirculation showed significantly increased *HTR1B* expression in PBMC compared to non‐thrombotic MPNs, non‐MPN thrombotic cases and normal subjects. Thrombotic MPN patients carried almost exclusively *JAK2* V617F mutation (90.6% of cases), this finding being consistent with the previously established etiological role of this mutation in MPN‐associated thrombosis.^17^ However, due the particularity of case selection in our retrospective study, the difference concerning the frequency and mutational load of JAK2 V617F was not significantly different between thrombotic and non‐thrombotic MPNs.

Thrombotic events, both arterial and venous, are a major cause of morbidity and mortality in MPN patients.^18^ The pathogenesis of thrombosis in MNP is multifactorial, being the result of the complex interaction between activated blood cells, pro‐adhesive endothelium, components of the coagulation system and mediators of inflammation.^19^ In addition, signs of impaired microcirculation are attributed to the presence of microthrombi in the arterioles, with PLTs playing a key role in the inflammation of the endothelium.^19^ Among leukocytes, monocytes represent an important source of prothrombotic factors.^20^ The presence of the *JAK2* V617F MPN‐driver mutation is also an independent risk factor for thrombotic events in ET patients,^21^ and a mutational load above 50% is associated with an increased thrombotic risk in PV.^22^

PBMCs are composed mainly of T lymphocytes (~ 70%), but include also B lymphocytes (~ 15%), natural killer cells (NK, ~ 10%), monocytes (~ 5%) and dendritic cells (~ 1%).^23^ In MPN patients some of these cells are part of the neoplastic clone and carry the driver mutation. As *JAK2* V617F mutation is acquired at the HSC level, it is present in all myeloid cells (including monocytes). It can also be found in lymphoid cells, especially in B lymphocytes and NK cells, and much less often in T lymphocytes, in advanced stages of disease.^24^ Similarly, mutations in exon 9 *CALR* were also detected in lymphoid cells in some patients, but with a much lower allelic frequency, suggesting that these mutations do not confer the same proliferation advantage in lymphoid cells compared to myeloid cells.^25^

Regarding the physiological *HTR1B* expression, studies on PBMC obtained from healthy volunteers indicated the essential role of this receptor in the growth and proliferation of T lymphocytes.^26^ *HTR1B* is also expressed by immature dendritic cells, and receptor stimulation leads to the mobilization of intracellular Ca_2_^+^.^27^

MPNs are characterized by a chronic inflammatory syndrome associated with an increased production of various inflammatory cytokines that promote the so‐called MPN‐thromboinflammation.^18^ Mutual activation of neutrophils and PLTs that leads to thrombus formation have been documented in cardiovascular pathology, and serotonin is one of the soluble mediators that induces neutrophil recruitment at the site of inflammation.^28^ Monocytes are activated by PLTs, too, and are involved in immune‐thrombosis.^28^ Additionally, it was shown that platelet‐lymphocyte interaction might also be relevant for inflammation and thrombosis.^29^

In our study, to rule out the possibility of an increased *HTR1B* expression in lymphocytes of patients with thrombosis in general, unrelated to MPN, we analysed the receptor expression in mononuclear cells obtained from patients with recent thrombosis and no signs of MPN and absence of MPN phenotypic driver mutations. The results indicated that thrombotic‐MPN patients displayed significantly higher levels of *HTR1B* relative expression compared to patients with non‐MPN thrombosis. These findings suggest that cells from the MPN malignant clone might exhibit an upregulation of *HTR1B*.

To our best knowledge, *HTR1B* expression have not been investigated in MPNs. We acknowledge that our study is limited by the low number of patients, especially from the non‐MPN thrombosis group and more studies are required to determine which cells overexpress HTR1B and exactly how is this related to thrombosis. Given that prediction of thrombotic complications remains an unmet medical need, further exploration of the link between increased *HTR1B* expression in MPNs and thrombosis might lead to the identification of a novel biomarker, and if causative, a novel therapy target. It remains to be studied whether in AML and MDS and other cancers with *HTR1B over‐*expression thrombosis is more frequent as well as whether the same cells over‐express it like in MPNs.

To conclude, increased levels of *HTR1B* expression in mononuclear cells might be associated with thrombosis in MPN patients, but larger studies are needed to confirm these results, including also testing of the receptor expression at protein level.

## AUTHOR CONTRIBUTIONS

**Petruta Gurban:** Conceptualization (lead); data curation (equal); formal analysis (equal); investigation (lead); methodology (lead); writing – original draft (equal); writing – review and editing (equal). **Cristina Mambet:** Conceptualization (equal); data curation (equal); formal analysis (equal); investigation (equal); methodology (equal); visualization (equal); writing – original draft (equal); writing – review and editing (equal). **Anca Botezatu:** Data curation (equal); formal analysis (equal); investigation (equal); methodology (equal); visualization (equal); writing – original draft (equal); writing – review and editing (equal). **Laura G. Necula:** Formal analysis (equal); investigation (equal); methodology (equal); writing – review and editing (equal). **Lilia Matei:** Data curation (equal); formal analysis (equal); investigation (equal); methodology (equal); writing – review and editing (equal). **Ana Iulia Neagu:** Data curation (equal); formal analysis (equal); investigation (equal); methodology (equal); writing – review and editing (equal). **Ioana Madalina Pitica:** Data curation (equal); formal analysis (equal); investigation (equal); methodology (equal); writing – review and editing (equal). **Laura Denisa Dragu:** Data curation (equal); formal analysis (equal); investigation (equal); methodology (equal); writing – review and editing (equal). **Alina Nastasie Schulman:** Data curation (equal); formal analysis (equal); investigation (equal); methodology (equal); writing – review and editing (equal). **Marius Ataman:** Investigation (equal); methodology (equal); visualization (equal); writing – review and editing (equal). **Saviana Nedeianu:** Data curation (equal); formal analysis (equal); investigation (equal); methodology (equal); writing – review and editing (equal). **Mihaela Chivu‐Economescu:** Data curation (equal); formal analysis (equal); investigation (equal); methodology (equal); visualization (equal); writing – review and editing (equal). **Coralia Bleotu:** Data curation (equal); formal analysis (equal); investigation (equal); methodology (equal); writing – review and editing (equal). **Gabriela Anton:** Conceptualization (equal); supervision (equal); validation (equal); writing – original draft (equal); writing – review and editing (equal). **Carmen Cristina Diaconu:** Conceptualization (equal); funding acquisition (lead); methodology (equal); project administration (lead); resources (lead); supervision (lead); validation (equal); writing – original draft (equal); writing – review and editing (equal).

## CONFLICT OF INTEREST STATEMENT

PG, is employed by Cytogenomic Medical Laboratory, Bucharest, Romania. The authors declare no conflict of interest.

## Data Availability

The data that support the findings of this study are available from the corresponding author upon reasonable request.
